# Fat embolism syndrome with cerebral fat embolism through a patent foramen ovale: A case report

**DOI:** 10.1097/MD.0000000000020569

**Published:** 2020-06-12

**Authors:** Lijuan Yang, Jiafang Wu, Baojun Wang

**Affiliations:** Department of Neurology, Baotou Central Hospital, Baotou, Inner, Mongolia Municipality, China.

**Keywords:** cerebral fat embolism, embolization pathway, fat embolism syndrome, paradoxical embolism, patent foramen ovale

## Abstract

**Rationale:**

Fat embolism syndrome with cerebral fat embolism, rarely observed at our neurology department, is often associated with long bone fractures. Its diagnosis is based on medical history and supportive imaging data and is usually not difficult. However, its pathogenesis remains poorly understood.

**Patient concerns:**

A 46-year-old woman was urgently presented to a nearby hospital because of a femur fracture caused by an accident. She rapidly developed somnolence and was suspected to have fat embolism syndrome.

**Diagnoses:**

Due to patients history of trauma and supportive imaging data, she was diagnosed with fat embolism syndrome obviously. However, severe brain damage confused our understanding of the pathogenesis. The subsequent diagnosis of fat embolism syndrome with patent foramen ovale provided a reasonable explanation.

**Interventions:**

Initially, we did not consider the fact that the patient had developed fat embolism syndrome and thus designed a comprehensive treatment program for fat embolism syndrome. Then the routine cardiac and vascular ultrasound screening were followed up, but patent foramen ovale was diagnosed unexpectedly, which led to a more aggressive treatment of brain injury.

**Outcomes:**

After relevant symptomatic treatment continued for nearly 3 months, an overall improvement was observed. Patients consciousness was restored but language disorders were left.

**Lessons:**

Clinicians should consider patent foramen ovale as the embolization pathway, particularly in young and middle-aged patients with cerebral embolism because it is often mistaken for a rare situation.

## 1 Introduction

Fat embolism syndrome (FES) is a rare condition and is associated with long bone fractures and plastic surgery. A 10-year retrospective study has shown that the incidence of FES with long-bone fracture was 0.9%.^[1]^ In recent years, the incidence of FES has been increasing with the increasing number of medical procedures such as plastic surgery.^[2–5]^ At present, there is still no gold standard for FES, with lack of relevant laboratory tests.^[6]^ The diagnosis of FES is an exclusion and depends mainly on clinic manifestations,^[6,7]^ which is characterized by hypoxemia, neurological impairment, and petechial rashes.^[7–11]^ Despite extensive literature and reports, FES with cerebral fat embolism(CFE) through a patent foramen ovale (PFO) is rarely observed in clinical practice.^[12,13]^ Here we present the rare case of a patient who developed FES 5 hours after trauma along with a demonstrable PFO.

## 2 Case report

**Figure 1:**
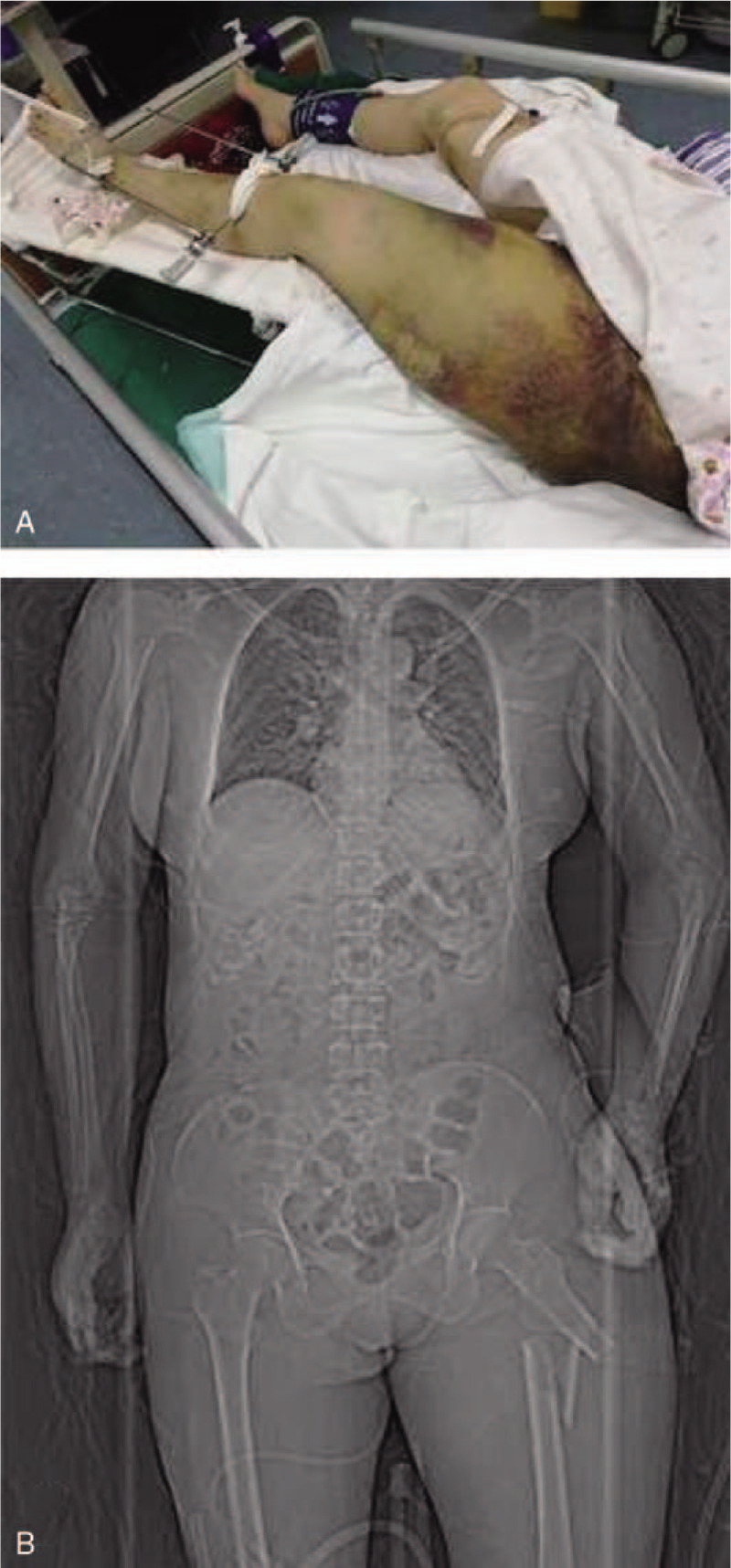
The left femur was fixed by external fixation (A); CT scan of the left femur (B) showing a comminuted fracture.

**Figure 2:**
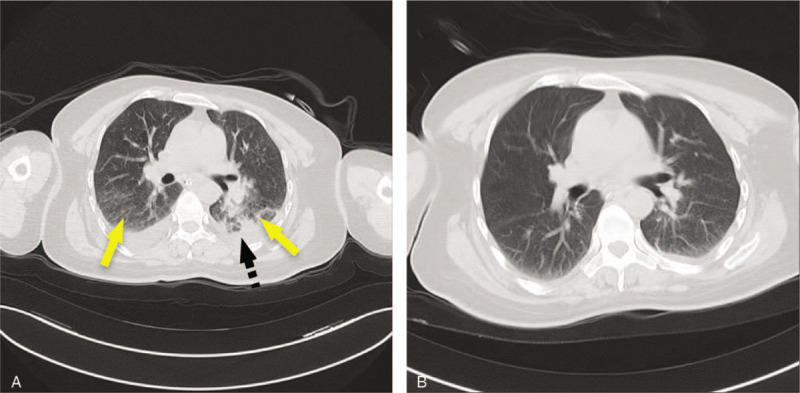
Axial nonenhanced lung CT: (A) showing bilateral involvement with excavation (black arrow) and pleural effusion (dashed arrow) (B) showing improved lesions.

**Figure 3:**
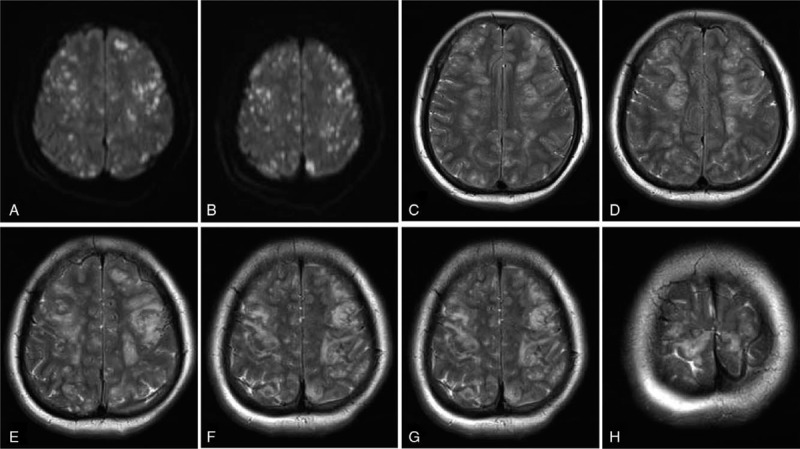
Diffusion-weighted (DW) MRI imaging (A, B) demonstrates multiple punctate foci of diffusion restriction in the gray-white junction. T2-weighted axial MRI (C–H) shows bilateral hyperintenseareas in the bilateral centrum semiovale and subcortical white matter. Images obtained 2 days after the femur fracture.

**Figure 4:**
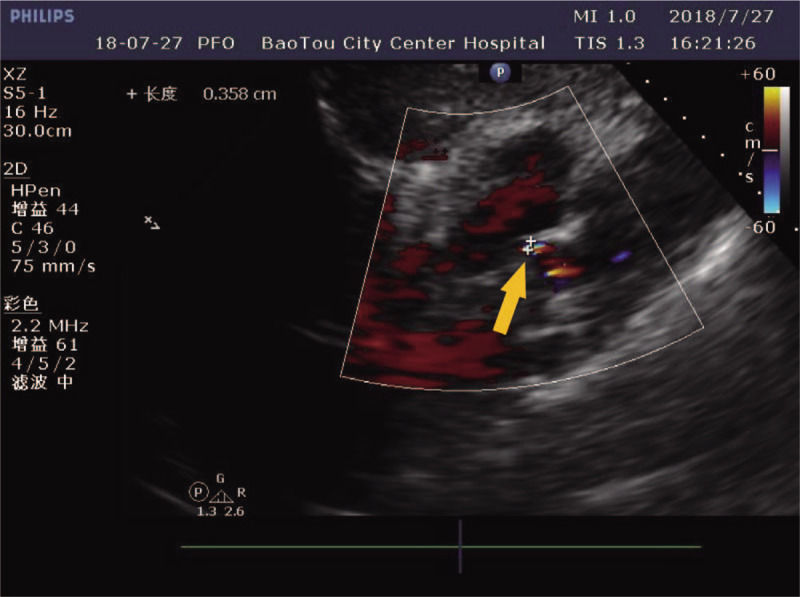
Transthoracic echocardiography showing shunt from PFO (yellow arrow). Images obtained 6 days after the femur fracture.

A 46-year-old woman was urgently presented to a nearby hospital because of a femur fracture caused by an accident. Although she was alert on arrival, she began to experience sleepiness approximately 5 hours later. A follow-up helical computed tomography (CT) of the brain revealed the absence of any additional severe lesions or skull fractures. Chest CT confirmed an exudative lesion, and a pulmonary vascular computed tomography angiography (CTA) revealed no obvious abnormalities. Based on the clinical and imaging findings, the patient was suspected to have CFE. After 2 days, the patient was moved to our neurology department to receive the appropriate care. Immediate physical examination on arrival revealed a Glasgow Coma Scale score of 4, a positive bilateral Babinski sign, a positive Kernig sign, and neck stiffness. The patients left thigh presented bruising and swelling and had been fixed with external fixation with restrained movement in the previous hospital (Fig. 1A). She presented an arterial pressure of 148/80 mm Hg, heart rate of 124 bpm, and partial pressure of oxygen (PO_2_) of 64 mm Hg. Her blood gas analysis revealed a pH value of 7.4. Total body CT revealed a left femoral comminuted fracture with dislocation (Fig. 1B). Her bilateral lung presented an exudative lesion as well as pleural effusion (Fig. 2A). Brain magnetic resonance imaging (MRI) revealed diffuse abnormal signals in the bilateral cerebral hemisphere, brainstem, and cerebellum, which were suggestive of embolization (Fig. 3). Combined with the patients trauma history and imaging findings of the lung and brain, the patient was initially diagnosed with FES, whose corresponding treatment is mainly supportive. Regarding the closed fracture of the left femur, the external fixation that had been performed at the previous hospital was appropriate, and no further treatment was applied. Subsequent cardiovascular screening, including echocardiography, cervical artery ultrasound and bilateral lower extremity vascular ultrasound, was performed, and all the findings were negative. Four days after the femur fracture, the patient presented with moderate dyspnea, and her arterial blood gas analysis retrieved a pH value of 7.4. PO_2_ was 35 mm Hg, partial pressure of carbon dioxide was 126 mm Hg, and arterial oxygen saturation was 97.5% under nasal oxygen. Fourteen days after the fracture, both the lung symptoms and lung CT findings were considerably improved (Fig. 2B). Transthoracic echocardiography ultrasound was performed again, and shunts through a PFO were observed (Fig. 4). On the following day, there was a gradual improvement in consciousness. However, the patients speech indicated dysphasia. Under active supportive care and hormone therapy, the patient was finally discharged with dysphasia and an untreated closed fracture.

## 3 Discussion

FES is a multisystemic disorder, and its pathogenesis remains unclear to date.^[6]^ However, 2 relatively well-accepted pathophysiological mechanisms have been indicated. One is the mechanical mechanism, which suggests that increased intramedullary pressure forces the bone marrow into injured venous sinusoids, thereby forming the fat emboli in the blood system.^[14]^ The other is the biochemical mechanism, which suggests that stress responses promote the conversion of stable fat particles in the blood into glycerol and toxic-free fatty acids, which occlude pulmonary vessels, thereby damaging pneumocytes and pulmonary endothelial cells and leading to vasogenic and cytotoxic edema with further respiratory symptoms, through which stable fat components in the blood vessels are transformed into fat emboli embolizating systemic vessels.^[15]^ The biochemical doctrine supports the occurrence of non-traumatic FES. As stated previously, fat emboli can be categorized into either endogenous or exogenous. Endogenous emboli originate from fat components in blood vessels, whereas exogenous emboli enter the vessel, which can originate from bone marrow or damaged fat in the liver or from plastic surgery.^[16–18]^ Regarding the fat embolization pathways, they can be divided into physiological and pathological. Fat emboli in the venous lumen first arrive to the pulmonary vascular system, further occluding it and causing respiratory symptoms. Some smaller fat emboli can pass via pulmonary circulation into the systemic circulation, thereby causing systemic embolic symptoms.^[11]^ Studies in animal models have shown that some emboli may enter the systemic circulation through the pulmonary capillary bed, causing systemic embolic symptoms.^[19]^ In related animal studies, oil red O staining was performed on rats lungs, kidneys, and brains 24 to 96 hours after FES, which revealed the presence of lipid droplets in and around the blood vessels, with maximum distribution in the lungs.^[20,21]^ The fact that lung damage is the most common in FES may be the physiological base. Occasionally, fat emboli can directly arrive into the systemic circulation system through intrapulmonary or intracardiac shunts. However, the most common pathological shunt pathway is frequently indemonstrable.

Although, there have been many reports of FES to date, its precise mechanism remains to be understood. An increasing number of reports have indicated that T2∗ sequences can serve as a pathognomonic feature of CFE and that the starfield pattern on diffusion-weighted MRI imaging is also characteristic of CFE in the context of long-bone fractures.^[22–25]^ However, pulmonary characteristic imaging findings are generally absent, and the most common findings are ground-glass opacities and dependent consolidations, associated with other variably overlapping signs, such as lobular opacities, random nodules, septal thickening, and bronchial wall thickening.^[26]^ In the present case report, a previously healthy patient presented with brain and lung symptoms following a femur fracture, together with typical brain and lung imaging findings. Thus, the patient was diagnosed with FES. However, we consider other diagnoses due to mild lung injury and severe brain lesions and subsequent examinations confirmed our suspicion. The patient presented with a right-to-left shunt through a PFO. Therefore, we considered that the patient was more likely to have paradox embolism than FES because fat emboli are mostly associated with the pathological pathway, with only a few or none originating from the physiological shunt pathways. Regarding the pulmonary lesions, the fat emboli were all from the physiological shunt pathway. Considering the limited scope and course of lung lesions, we believe that there are not many fat emboli entering the lungs and that they may be partly cleared by the lungs, which has been demonstrated in animal studies.^[27]^ Notwithstanding the relative rarity of this case, it offers valuable insights into FES. The continuous improvements in diagnostic techniques and intensive research will provide a clear embolic approach specific to each affected organ, similar to the case reported in the present study.

## Acknowledgments

In this case report, no special individuals and organizations need to be appreciated.

## Author contributions

**Conceptualization:** Lijuan Yang, Baojun Wang.

**Data curation:** Lijuan Yang, Jiafang Wu.

**Formal analysis:** Jiafang Wu.

**Methodology:** Baojun Wang.

**Resources:** Jiafang Wu.

**Writing – original draft:** Baojun Wang.

**Writing – review & editing:** Lijuan Yang, Baojun Wang.
